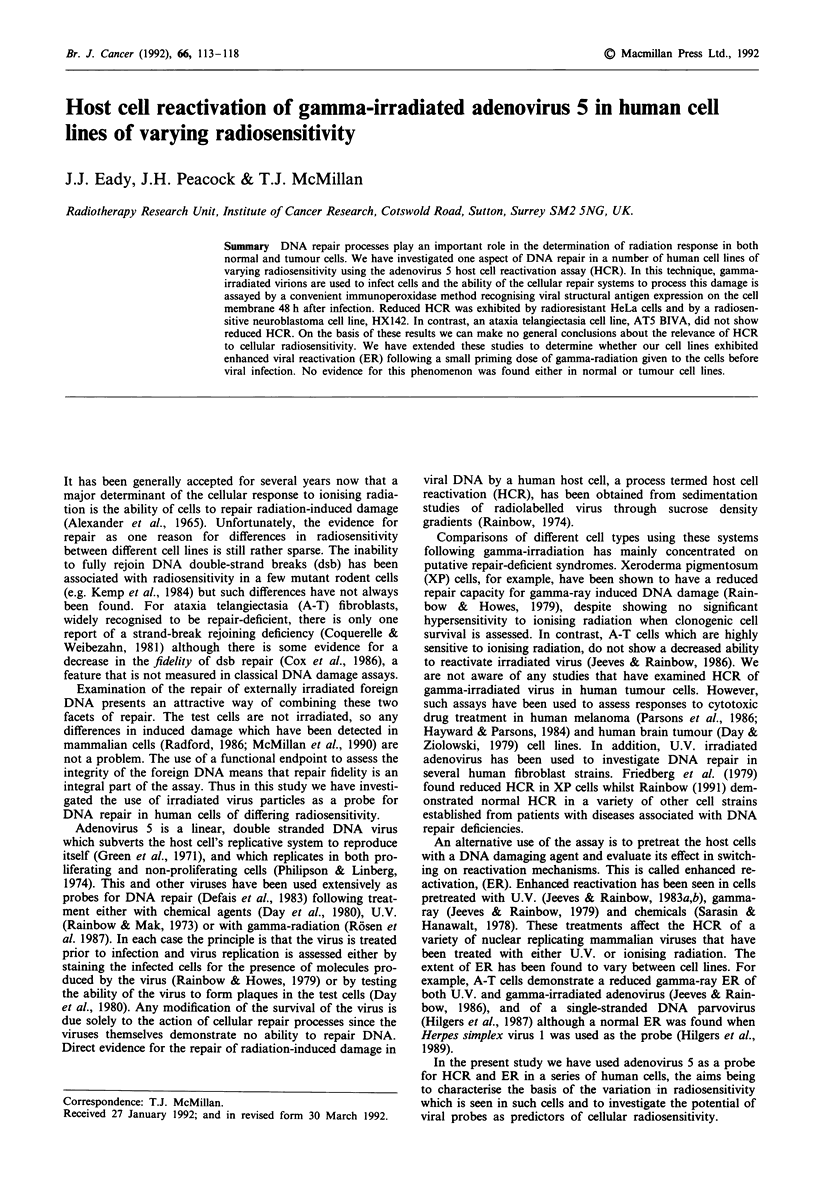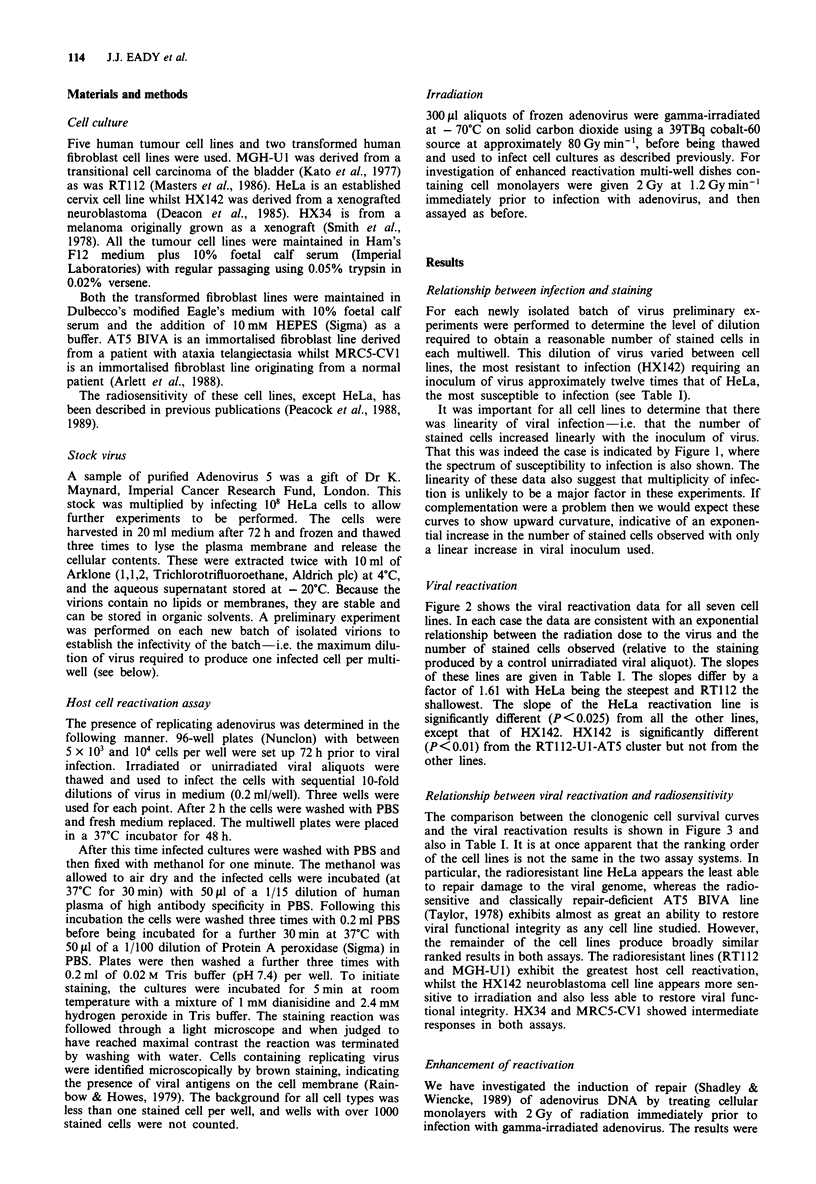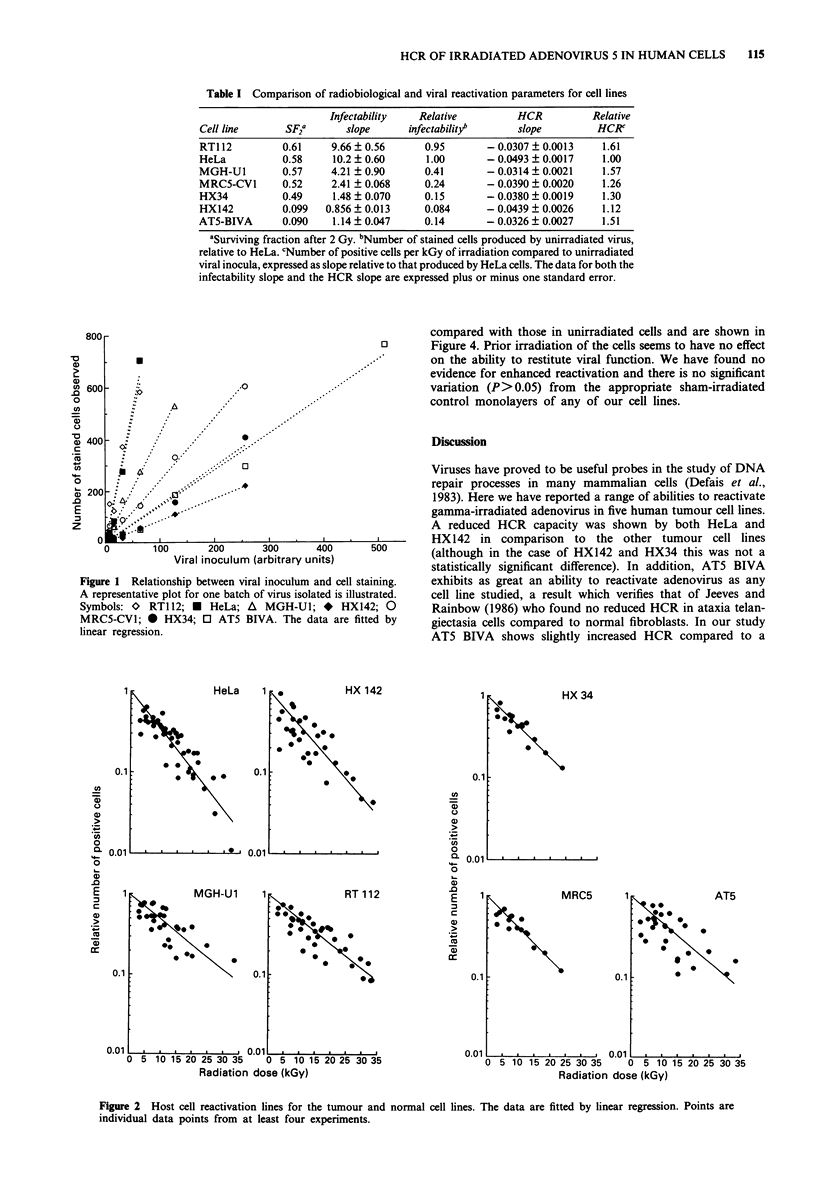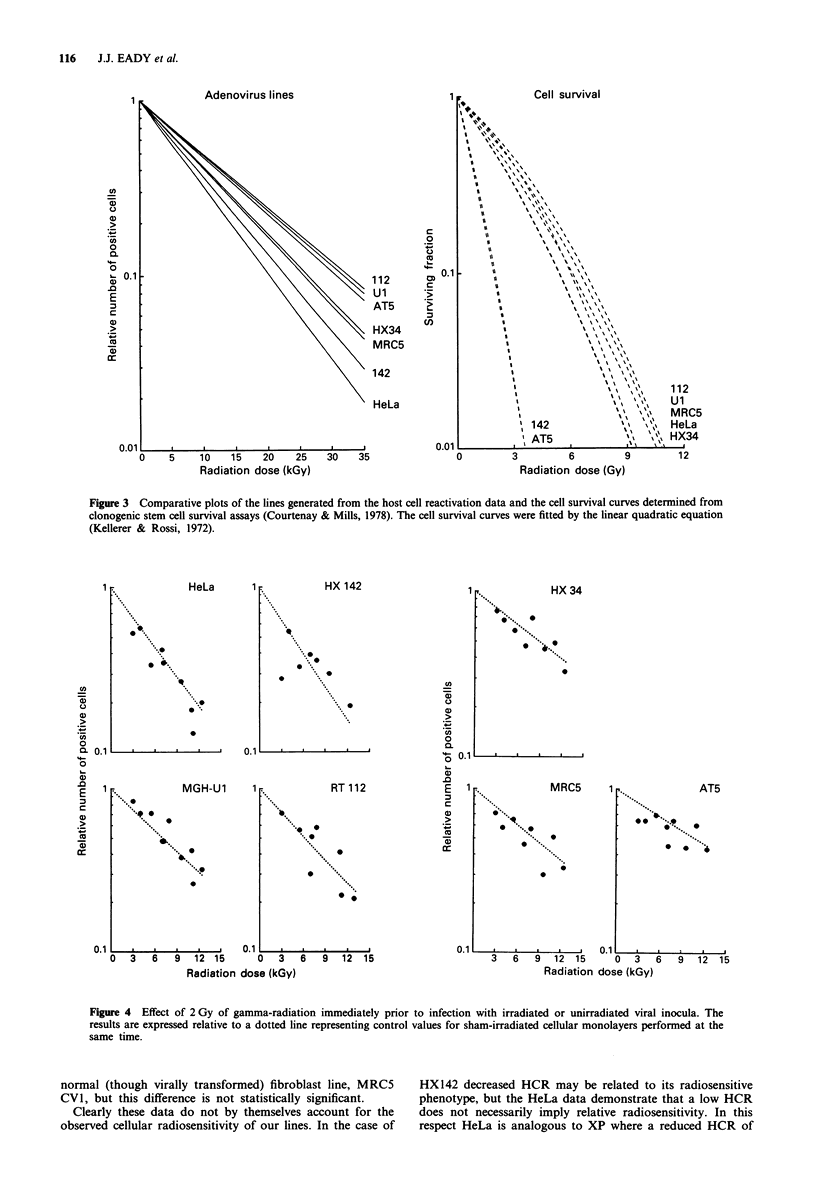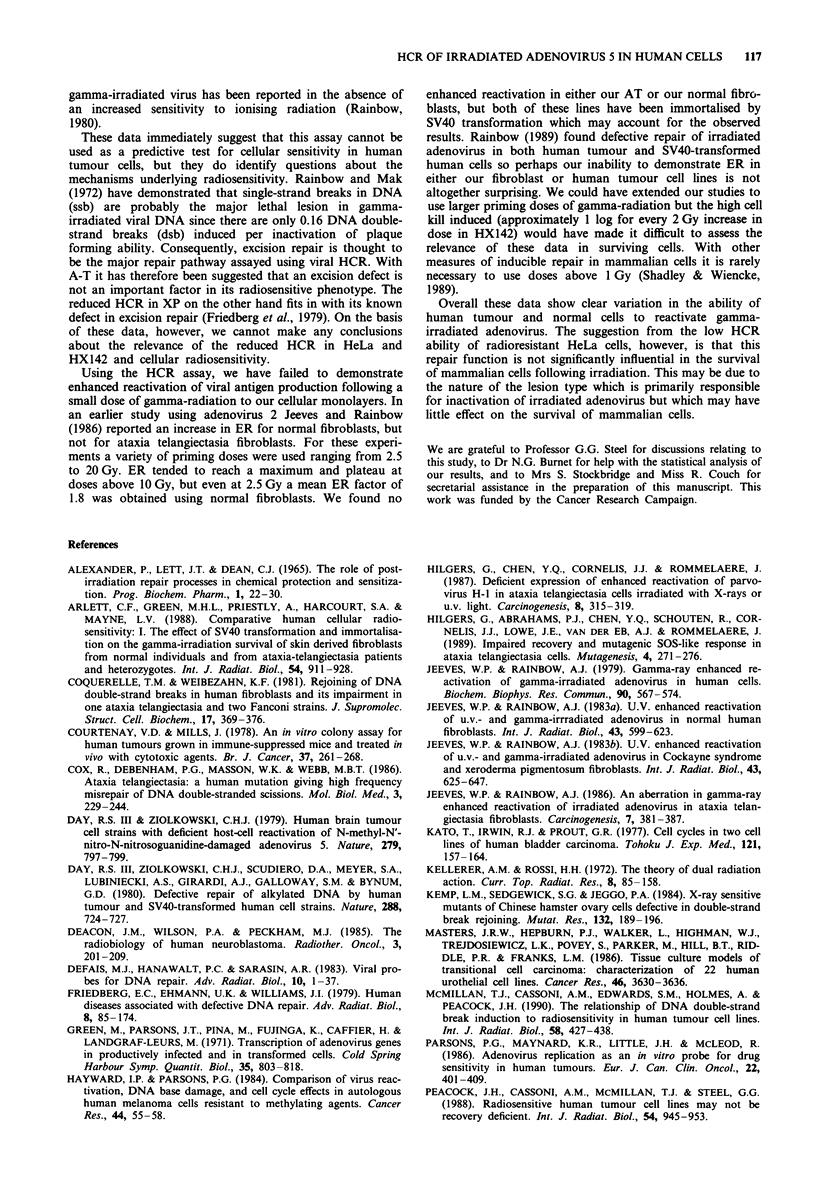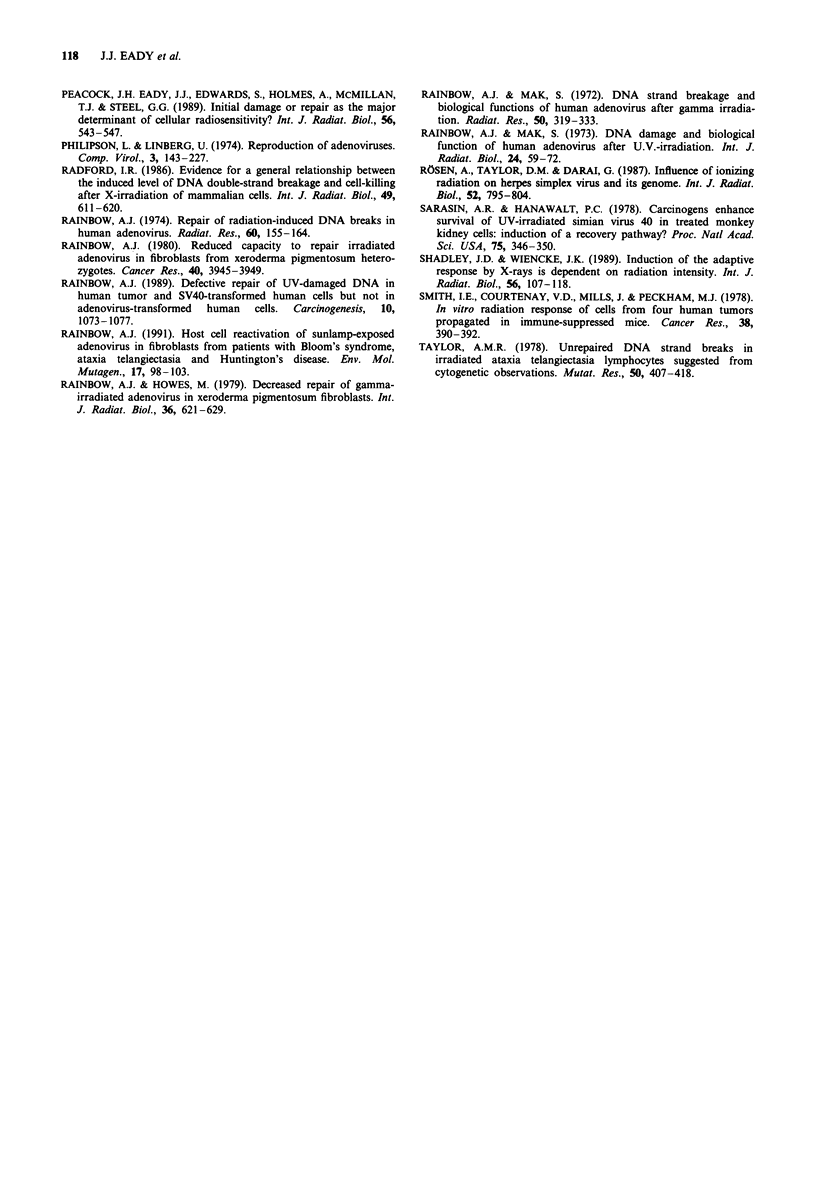# Host cell reactivation of gamma-irradiated adenovirus 5 in human cell lines of varying radiosensitivity.

**DOI:** 10.1038/bjc.1992.226

**Published:** 1992-07

**Authors:** J. J. Eady, J. H. Peacock, T. J. McMillan

**Affiliations:** Radiotherapy Research Unit, Institute of Cancer Research, Sutton, Surrey, UK.

## Abstract

DNA repair processes play an important role in the determination of radiation response in both normal and tumour cells. We have investigated one aspect of DNA repair in a number of human cell lines of varying radiosensitivity using the adenovirus 5 host cell reactivation assay (HCR). In this technique, gamma-irradiated virions are used to infect cells and the ability of the cellular repair systems to process this damage is assayed by a convenient immunoperoxidase method recognising viral structural antigen expression on the cell membrane 48 h after infection. Reduced HCR was exhibited by radioresistant HeLa cells and by a radiosensitive neuroblastoma cell line, HX142. In contrast, an ataxia telangiectasia cell line, AT5 BIVA, did not show reduced HCR. On the basis of these results we can make no general conclusions about the relevance of HCR to cellular radiosensitivity. We have extended these studies to determine whether our cell lines exhibited enhanced viral reactivation (ER) following a small priming dose of gamma-radiation given to the cells before viral infection. No evidence for this phenomenon was found either in normal or tumour cell lines.


*Br. J. Cancer* (1992), **66**, 113–118 © Macmillan Press Ltd., 1992

### Host cell reactivation of gamma-irradiated adenovirus 5 in human cell lines of varying radiosensitivity

J.J. Eady, J.H. Peacock & T.J. McMillan

*Radiotherapy Research Unit, Institute of Cancer Research, Cotswold Road, Sutton, Surrey SM2 5NG, UK.*

**Summary** DNA repair processes play an important role in the determination of radiation response in both normal and tumour cells. We have investigated one aspect of DNA repair in a number of human cell lines of varying radiosensitivity using the adenovirus 5 host cell reactivation assay (HCR). In this technique, gamma-irradiated virions are used to infect cells and the ability of the cellular repair systems to process this damage is assayed by a convenient immunoperoxidase method recognising viral structural antigen expression on the cell membrane 48 h after infection. Reduced HCR was exhibited by radioresistant HeLa cells and by a radiosensitive neuroblastoma cell line, HX142. In contrast, an ataxia telangiectasia cell line, AT5 BIVA, did not show reduced HCR. On the basis of these results we can make no general conclusions about the relevance of HCR to cellular radiosensitivity. We have extended these studies to determine whether our cell lines exhibited enhanced viral reactivation (ER) following a small priming dose of gamma-radiation given to the cells before viral infection. No evidence for this phenomenon was found either in normal or tumour cell lines.

It has been generally accepted for several years now that a major determinant of the cellular response to ionising radiation is the ability of cells to repair radiation-induced damage (Alexander *et al.*, 1965). Unfortunately, the evidence for repair as one reason for differences in radiosensitivity between different cell lines is still rather sparse. The inability to fully rejoin DNA double-strand breaks (dsb) has been associated with radiosensitivity in a few mutant rodent cells (e.g. Kemp *et al.*, 1984) but such differences have not always been found. For ataxia telangiectasia (A-T) fibroblasts, widely recognised to be repair-deficient, there is only one report of a strand-break rejoining deficiency (Coquerelle & Weibezahn, 1981) although there is some evidence for a decrease in the *fidelity* of dsb repair (Cox *et al.*, 1986), a feature that is not measured in classical DNA damage assays.

Examination of the repair of externally irradiated foreign DNA presents an attractive way of combining these two facets of repair. The test cells are not irradiated, so any differences in induced damage which have been detected in mammalian cells (Radford, 1986; McMillan *et al.*, 1990) are not a problem. The use of a functional endpoint to assess the integrity of the foreign DNA means that repair fidelity is an integral part of the assay. Thus in this study we have investigated the use of irradiated virus particles as a probe for DNA repair in human cells of differing radiosensitivity.

Adenovirus 5 is a linear, double stranded DNA virus which subverts the host cell's replicative system to reproduce itself (Green *et al.*, 1971), and which replicates in both proliferating and non-proliferating cells (Philipson & Linberg, 1974). This and other viruses have been used extensively as probes for DNA repair (Defais *et al.*, 1983) following treatment either with chemical agents (Day *et al.*, 1980), U.V. (Rainbow & Mak, 1973) or with gamma-radiation (Rösen *et al.* 1987). In each case the principle is that the virus is treated prior to infection and virus replication is assessed either by staining the infected cells for the presence of molecules produced by the virus (Rainbow & Howes, 1979) or by testing the ability of the virus to form plaques in the test cells (Day *et al.*, 1980). Any modification of the survival of the virus is due solely to the action of cellular repair processes since the viruses themselves demonstrate no ability to repair DNA. Direct evidence for the repair of radiation-induced damage in viral DNA by a human host cell, a process termed host cell reactivation (HCR), has been obtained from sedimentation studies of radiolabelled virus through sucrose density gradients (Rainbow, 1974).

Comparisons of different cell types using these systems following gamma-irradiation has mainly concentrated on putative repair-deficient syndromes. Xeroderma pigmentosum (XP) cells, for example, have been shown to have a reduced repair capacity for gamma-ray induced DNA damage (Rainbow & Howes, 1979), despite showing no significant hypersensitivity to ionising radiation when clonogenic cell survival is assessed. In contrast, A-T cells which are highly sensitive to ionising radiation, do not show a decreased ability to reactivate irradiated virus (Jeeves & Rainbow, 1986). We are not aware of any studies that have examined HCR of gamma-irradiated virus in human tumour cells. However, such assays have been used to assess responses to cytotoxic drug treatment in human melanoma (Parsons *et al.*, 1986; Hayward & Parsons, 1984) and human brain tumour (Day & Ziolowski, 1979) cell lines. In addition, U.V. irradiated adenovirus has been used to investigate DNA repair in several human fibroblast strains. Friedberg *et al.* (1979) found reduced HCR in XP cells whilst Rainbow (1991) demonstrated normal HCR in a variety of other cell strains established from patients with diseases associated with DNA repair deficiencies.

An alternative use of the assay is to pretreat the host cells with a DNA damaging agent and evaluate its effect in switching on reactivation mechanisms. This is called enhanced reactivation, (ER). Enhanced reactivation has been seen in cells pretreated with U.V. (Jeeves & Rainbow, 1983*a*,*b*), gamma-ray (Jeeves & Rainbow, 1979) and chemicals (Sarasin & Hanawalt, 1978). These treatments affect the HCR of a variety of nuclear replicating mammalian viruses that have been treated with either U.V. or ionising radiation. The extent of ER has been found to vary between cell lines. For example, A-T cells demonstrate a reduced gamma-ray ER of both U.V. and gamma-irradiated adenovirus (Jeeves & Rainbow, 1986), and of a single-stranded DNA parvovirus (Hilgers *et al.*, 1987) although a normal ER was found when *Herpes simplex* virus 1 was used as the probe (Hilgers *et al.*, 1989).

In the present study we have used adenovirus 5 as a probe for HCR and ER in a series of human cells, the aims being to characterise the basis of the variation in radiosensitivity which is seen in such cells and to investigate the potential of viral probes as predictors of cellular radiosensitivity.

Correspondence: T.J. McMillan.
Received 27 January 1992; and in revised form 30 March 1992.

### Materials and methods

### Cell culture

Five human tumour cell lines and two transformed human fibroblast cell lines were used. MGH-U1 was derived from a transitional cell carcinoma of the bladder (Kato *et al.*, 1977) as was RT112 (Masters *et al.*, 1986). HeLa is an established cervix cell line whilst HX142 was derived from a xenografted neuroblastoma (Deacon *et al.*, 1985). HX34 is from a melanoma originally grown as a xenograft (Smith *et al.*, 1978). All the tumour cell lines were maintained in Ham's F12 medium plus 10% foetal calf serum (Imperial Laboratories) with regular passaging using 0.05% trypsin in 0.02% versene.

Both the transformed fibroblast lines were maintained in Dulbecco's modified Eagle's medium with 10% foetal calf serum and the addition of 10 mM HEPES (Sigma) as a buffer. AT5 BIVA is an immortalised fibroblast line derived from a patient with ataxia telangiectasia whilst MRC5-CV1 is an immortalised fibroblast line originating from a normal patient (Arlett *et al.*, 1988).

The radiosensitivity of these cell lines, except HeLa, has been described in previous publications (Peacock *et al.*, 1988, 1989).

### Stock virus

A sample of purified Adenovirus 5 was a gift of Dr K. Maynard, Imperial Cancer Research Fund, London. This stock was multiplied by infecting $10^8$ HeLa cells to allow further experiments to be performed. The cells were harvested in 20 ml medium after 72 h and frozen and thawed three times to lyse the plasma membrane and release the cellular contents. These were extracted twice with 10 ml of Arklone (1,1,2, Trichlorotrifluoroethane, Aldrich plc) at 4°C, and the aqueous supernatant stored at − 20°C. Because the virions contain no lipids or membranes, they are stable and can be stored in organic solvents. A preliminary experiment was performed on each new batch of isolated virions to establish the infectivity of the batch—i.e. the maximum dilution of virus required to produce one infected cell per multiwell (see below).

### Host cell reactivation assay

The presence of replicating adenovirus was determined in the following manner. 96-well plates (Nunclon) with between $5 \times 10^3$ and $10^4$ cells per well were set up 72 h prior to viral infection. Irradiated or unirradiated viral aliquots were thawed and used to infect the cells with sequential 10-fold dilutions of virus in medium (0.2 ml/well). Three wells were used for each point. After 2 h the cells were washed with PBS and fresh medium replaced. The multiwell plates were placed in a 37°C incubator for 48 h.

After this time infected cultures were washed with PBS and then fixed with methanol for one minute. The methanol was allowed to air dry and the infected cells were incubated (at 37°C for 30 min) with 50 µl of a 1/15 dilution of human plasma of high antibody specificity in PBS. Following this incubation the cells were washed three times with 0.2 ml PBS before being incubated for a further 30 min at 37°C with 50 µl of a 1/100 dilution of Protein A peroxidase (Sigma) in PBS. Plates were then washed a further three times with 0.2 ml of 0.02 M Tris buffer (pH 7.4) per well. To initiate staining, the cultures were incubated for 5 min at room temperature with a mixture of 1 mM dianisidine and 2.4 mM hydrogen peroxide in Tris buffer. The staining reaction was followed through a light microscope and when judged to have reached maximal contrast the reaction was terminated by washing with water. Cells containing replicating virus were identified microscopically by brown staining, indicating the presence of viral antigens on the cell membrane (Rainbow & Howes, 1979). The background for all cell types was less than one stained cell per well, and wells with over 1000 stained cells were not counted.

### Irradiation

300 µl aliquots of frozen adenovirus were gamma-irradiated at − 70°C on solid carbon dioxide using a 39TBq cobalt-60 source at approximately 80 Gy min$^{-1}$, before being thawed and used to infect cell cultures as described previously. For investigation of enhanced reactivation multi-well dishes containing cell monolayers were given 2 Gy at 1.2 Gy min$^{-1}$ immediately prior to infection with adenovirus, and then assayed as before.

### Results

### Relationship between infection and staining

For each newly isolated batch of virus preliminary experiments were performed to determine the level of dilution required to obtain a reasonable number of stained cells in each multiwell. This dilution of virus varied between cell lines, the most resistant to infection (HX142) requiring an inoculum of virus approximately twelve times that of HeLa, the most susceptible to infection (see Table I).

It was important for all cell lines to determine that there was linearity of viral infection—i.e. that the number of stained cells increased linearly with the inoculum of virus. That this was indeed the case is indicated by Figure 1, where the spectrum of susceptibility to infection is also shown. The linearity of these data also suggest that multiplicity of infection is unlikely to be a major factor in these experiments. If complementation were a problem then we would expect these curves to show upward curvature, indicative of an exponential increase in the number of stained cells observed with only a linear increase in viral inoculum used.

### Viral reactivation

Figure 2 shows the viral reactivation data for all seven cell lines. In each case the data are consistent with an exponential relationship between the radiation dose to the virus and the number of stained cells observed (relative to the staining produced by a control unirradiated viral aliquot). The slopes of these lines are given in Table I. The slopes differ by a factor of 1.61 with HeLa being the steepest and RT112 the shallowest. The slope of the HeLa reactivation line is significantly different ($P < 0.025$) from all the other lines, except that of HX142. HX142 is significantly different ($P < 0.01$) from the RT112-U1-AT5 cluster but not from the other lines.

### Relationship between viral reactivation and radiosensitivity

The comparison between the clonogenic cell survival curves and the viral reactivation results is shown in Figure 3 and also in Table I. It is at once apparent that the ranking order of the cell lines is not the same in the two assay systems. In particular, the radioresistant line HeLa appears the least able to repair damage to the viral genome, whereas the radiosensitive and classically repair-deficient AT5 BIVA line (Taylor, 1978) exhibits almost as great an ability to restore viral functional integrity as any cell line studied. However, the remainder of the cell lines produce broadly similar ranked results in both assays. The radioresistant lines (RT112 and MGH-U1) exhibit the greatest host cell reactivation, whilst the HX142 neuroblastoma cell line appears more sensitive to irradiation and also less able to restore viral functional integrity. HX34 and MRC5-CV1 showed intermediate responses in both assays.

### Enhancement of reactivation

We have investigated the induction of repair (Shadley & Wiencke, 1989) of adenovirus DNA by treating cellular monolayers with 2 Gy of radiation immediately prior to infection with gamma-irradiated adenovirus. The results were

**Table I** Comparison of radiobiological and viral reactivation parameters for cell lines

| *Cell line* | $SF_2$[a] | *Infectability slope* | *Relative infectability*[b] | *HCR slope* | *Relative HCR*[c] |
|---|---|---|---|---|---|
| RT112 | 0.61 | 9.66 ± 0.56 | 0.95 | − 0.0307 ± 0.0013 | 1.61 |
| HeLa | 0.58 | 10.2 ± 0.60 | 1.00 | − 0.0493 ± 0.0017 | 1.00 |
| MGH-U1 | 0.57 | 4.21 ± 0.90 | 0.41 | − 0.0314 ± 0.0021 | 1.57 |
| MRC5-CV1 | 0.52 | 2.41 ± 0.068 | 0.24 | − 0.0390 ± 0.0020 | 1.26 |
| HX34 | 0.49 | 1.48 ± 0.070 | 0.15 | − 0.0380 ± 0.0019 | 1.30 |
| HX142 | 0.099 | 0.856 ± 0.013 | 0.084 | − 0.0439 ± 0.0026 | 1.12 |
| AT5-BIVA | 0.090 | 1.14 ± 0.047 | 0.14 | − 0.0326 ± 0.0027 | 1.51 |

[a]Surviving fraction after 2 Gy. [b]Number of stained cells produced by unirradiated virus, relative to HeLa. [c]Number of positive cells per kGy of irradiation compared to unirradiated viral inocula, expressed as slope relative to that produced by HeLa cells. The data for both the infectability slope and the HCR slope are expressed plus or minus one standard error.

**Figure 1** Relationship between viral inoculum and cell staining. A representative plot for one batch of virus isolated is illustrated. Symbols: ◇ RT112; ■ HeLa; △ MGH-U1; ◆ HX142; ○ MRC5-CV1; ● HX34; □ AT5 BIVA. The data are fitted by linear regression.

compared with those in unirradiated cells and are shown in Figure 4. Prior irradiation of the cells seems to have no effect on the ability to restitute viral function. We have found no evidence for enhanced reactivation and there is no significant variation ($P > 0.05$) from the appropriate sham-irradiated control monolayers of any of our cell lines.

### Discussion

Viruses have proved to be useful probes in the study of DNA repair processes in many mammalian cells (Defais *et al.*, 1983). Here we have reported a range of abilities to reactivate gamma-irradiated adenovirus in five human tumour cell lines. A reduced HCR capacity was shown by both HeLa and HX142 in comparison to the other tumour cell lines (although in the case of HX142 and HX34 this was not a statistically significant difference). In addition, AT5 BIVA exhibits as great an ability to reactivate adenovirus as any cell line studied, a result which verifies that of Jeeves and Rainbow (1986) who found no reduced HCR in ataxia telangiectasia cells compared to normal fibroblasts. In our study AT5 BIVA shows slightly increased HCR compared to a

**Figure 2** Host cell reactivation lines for the tumour and normal cell lines. The data are fitted by linear regression. Points are individual data points from at least four experiments.

**Figure 3** Comparative plots of the lines generated from the host cell reactivation data and the cell survival curves determined from clonogenic stem cell survival assays (Courtenay & Mills, 1978). The cell survival curves were fitted by the linear quadratic equation (Kellerer & Rossi, 1972).

**Figure 4** Effect of 2 Gy of gamma-radiation immediately prior to infection with irradiated or unirradiated viral inocula. The results are expressed relative to a dotted line representing control values for sham-irradiated cellular monolayers performed at the same time.

normal (though virally transformed) fibroblast line, MRC5 CV1, but this difference is not statistically significant.

Clearly these data do not by themselves account for the observed cellular radiosensitivity of our lines. In the case of HX142 decreased HCR may be related to its radiosensitive phenotype, but the HeLa data demonstrate that a low HCR does not necessarily imply relative radiosensitivity. In this respect HeLa is analogous to XP where a reduced HCR of gamma-irradiated virus has been reported in the absence of an increased sensitivity to ionising radiation (Rainbow, 1980).

These data immediately suggest that this assay cannot be used as a predictive test for cellular sensitivity in human tumour cells, but they do identify questions about the mechanisms underlying radiosensitivity. Rainbow and Mak (1972) have demonstrated that single-strand breaks in DNA (ssb) are probably the major lethal lesion in gamma-irradiated viral DNA since there are only 0.16 DNA double-strand breaks (dsb) induced per inactivation of plaque forming ability. Consequently, excision repair is thought to be the major repair pathway assayed using viral HCR. With A-T it has therefore been suggested that an excision defect is not an important factor in its radiosensitive phenotype. The reduced HCR in XP on the other hand fits in with its known defect in excision repair (Friedberg *et al.*, 1979). On the basis of these data, however, we cannot make any conclusions about the relevance of the reduced HCR in HeLa and HX142 and cellular radiosensitivity.

Using the HCR assay, we have failed to demonstrate enhanced reactivation of viral antigen production following a small dose of gamma-radiation to our cellular monolayers. In an earlier study using adenovirus 2 Jeeves and Rainbow (1986) reported an increase in ER for normal fibroblasts, but not for ataxia telangiectasia fibroblasts. For these experiments a variety of priming doses were used ranging from 2.5 to 20 Gy. ER tended to reach a maximum and plateau at doses above 10 Gy, but even at 2.5 Gy a mean ER factor of 1.8 was obtained using normal fibroblasts. We found no enhanced reactivation in either our AT or our normal fibroblasts, but both of these lines have been immortalised by SV40 transformation which may account for the observed results. Rainbow (1989) found defective repair of irradiated adenovirus in both human tumour and SV40-transformed human cells so perhaps our inability to demonstrate ER in either our fibroblast or human tumour cell lines is not altogether surprising. We could have extended our studies to use larger priming doses of gamma-radiation but the high cell kill induced (approximately 1 log for every 2 Gy increase in dose in HX142) would have made it difficult to assess the relevance of these data in surviving cells. With other measures of inducible repair in mammalian cells it is rarely necessary to use doses above 1 Gy (Shadley & Wiencke, 1989).

Overall these data show clear variation in the ability of human tumour and normal cells to reactivate gamma-irradiated adenovirus. The suggestion from the low HCR ability of radioresistant HeLa cells, however, is that this repair function is not significantly influential in the survival of mammalian cells following irradiation. This may be due to the nature of the lesion type which is primarily responsible for inactivation of irradiated adenovirus but which may have little effect on the survival of mammalian cells.

We are grateful to Professor G.G. Steel for discussions relating to this study, to Dr N.G. Burnet for help with the statistical analysis of our results, and to Mrs S. Stockbridge and Miss R. Couch for secretarial assistance in the preparation of this manuscript. This work was funded by the Cancer Research Campaign.

### References

ALEXANDER, P., LETT, J.T. & DEAN, C.J. (1965). The role of post-irradiation repair processes in chemical protection and sensitization. *Prog. Biochem. Pharm.*, **1**, 22–30.

ARLETT, C.F., GREEN, M.H.L., PRIESTLY, A., HARCOURT, S.A. & MAYNE, L.V. (1988). Comparative human cellular radiosensitivity: I. The effect of SV40 transformation and immortalisation on the gamma-irradiation survival of skin derived fibroblasts from normal individuals and from ataxia-telangiectasia patients and heterozygotes. *Int. J. Radiat. Biol.*, **54**, 911–928.

COQUERELLE, T.M. & WEIBEZAHN, K.F. (1981). Rejoining of DNA double-strand breaks in human fibroblasts and its impairment in one ataxia telangiectasia and two Fanconi strains. *J. Supromolec. Struct. Cell. Biochem.*, **17**, 369–376.

COURTENAY, V.D. & MILLS, J. (1978). An *in vitro* colony assay for human tumours grown in immune-suppressed mice and treated *in vivo* with cytotoxic agents. *Br. J. Cancer*, **37**, 261–268.

COX, R., DEBENHAM, P.G., MASSON, W.K. & WEBB, M.B.T. (1986). Ataxia telangiectasia: a human mutation giving high frequency misrepair of DNA double-stranded scissions. *Mol. Biol. Med.*, **3**, 229–244.

DAY, R.S. III & ZIOLKOWSKI, C.H.J. (1979). Human brain tumour cell strains with deficient host-cell reactivation of N-methyl-N'-nitro-N-nitrosoguanidine-damaged adenovirus 5. *Nature*, **279**, 797–799.

DAY, R.S. III, ZIOLKOWSKI, C.H.J., SCUDIERO, D.A., MEYER, S.A., LUBINIECKI, A.S., GIRARDI, A.J., GALLOWAY, S.M. & BYNUM, G.D. (1980). Defective repair of alkylated DNA by human tumour and SV40-transformed human cell strains. *Nature*, **288**, 724–727.

DEACON, J.M., WILSON, P.A. & PECKHAM, M.J. (1985). The radiobiology of human neuroblastoma. *Radiother. Oncol.*, **3**, 201–209.

DEFAIS, M.J., HANAWALT, P.C. & SARASIN, A.R. (1983). Viral probes for DNA repair. *Adv. Radiat. Biol.*, **10**, 1–37.

FRIEDBERG, E.C., EHMANN, U.K. & WILLIAMS, J.I. (1979). Human diseases associated with defective DNA repair. *Adv. Radiat. Biol.*, **8**, 85–174.

GREEN, M., PARSONS, J.T., PINA, M., FUJINGA, K., CAFFIER, H. & LANDGRAF-LEURS, M. (1971). Transcription of adenovirus genes in productively infected and in transformed cells. *Cold Spring Harbour Symp. Quantit. Biol.*, **35**, 803–818.

HAYWARD, I.P. & PARSONS, P.G. (1984). Comparison of virus reactivation, DNA base damage, and cell cycle effects in autologous human melanoma cells resistant to methylating agents. *Cancer Res.*, **44**, 55–58.

HILGERS, G., CHEN, Y.Q., CORNELIS, J.J. & ROMMELAERE, J. (1987). Deficient expression of enhanced reactivation of parvovirus H-1 in ataxia telangiectasia cells irradiated with X-rays or u.v. light. *Carcinogenesis*, **8**, 315–319.

HILGERS, G., ABRAHAMS, P.J., CHEN, Y.Q., SCHOUTEN, R., CORNELIS, J.J., LOWE, J.E., VAN DER EB, A.J. & ROMMELAERE, J. (1989). Impaired recovery and mutagenic SOS-like response in ataxia telangiectasia cells. *Mutagenesis*, **4**, 271–276.

JEEVES, W.P. & RAINBOW, A.J. (1979). Gamma-ray enhanced reactivation of gamma-irradiated adenovirus in human cells. *Biochem. Biophys. Res. Commun.*, **90**, 567–574.

JEEVES, W.P. & RAINBOW, A.J. (1983*a*). U.V. enhanced reactivation of u.v.- and gamma-irradiated adenovirus in normal human fibroblasts. *Int. J. Radiat. Biol.*, **43**, 599–623.

JEEVES, W.P. & RAINBOW, A.J. (1983*b*). U.V. enhanced reactivation of u.v.- and gamma-irradiated adenovirus in Cockayne syndrome and xeroderma pigmentosum fibroblasts. *Int. J. Radiat. Biol.*, **43**, 625–647.

JEEVES, W.P. & RAINBOW, A.J. (1986). An aberration in gamma-ray enhanced reactivation of irradiated adenovirus in ataxia telangiectasia fibroblasts. *Carcinogenesis*, **7**, 381–387.

KATO, T., IRWIN, R.J. & PROUT, G.R. (1977). Cell cycles in two cell lines of human bladder carcinoma. *Tohoku J. Exp. Med.*, **121**, 157–164.

KELLERER, A.M. & ROSSI, H.H. (1972). The theory of dual radiation action. *Curr. Top. Radiat. Res.*, **8**, 85–158.

KEMP, L.M., SEDGEWICK, S.G. & JEGGO, P.A. (1984). X-ray sensitive mutants of Chinese hamster ovary cells defective in double-strand break rejoining. *Mutat. Res.*, **132**, 189–196.

MASTERS, J.R.W., HEPBURN, P.J., WALKER, L., HIGHMAN, W.J., TREJDOSIEWICZ, L.K., POVEY, S., PARKER, M., HILL, B.T., RIDDLE, P.R. & FRANKS, L.M. (1986). Tissue culture models of transitional cell carcinoma: characterization of 22 human urothelial cell lines. *Cancer Res.*, **46**, 3630–3636.

MCMILLAN, T.J., CASSONI, A.M., EDWARDS, S.M., HOLMES, A. & PEACOCK, J.H. (1990). The relationship of DNA double-strand break induction to radiosensitivity in human tumour cell lines. *Int. J. Radiat. Biol.*, **58**, 427–438.

PARSONS, P.G., MAYNARD, K.R., LITTLE, J.H. & MCLEOD, R. (1986). Adenovirus replication as an *in vitro* probe for drug sensitivity in human tumours. *Eur. J. Can. Clin. Oncol.*, **22**, 401–409.

PEACOCK, J.H., CASSONI, A.M., MCMILLAN, T.J. & STEEL, G.G. (1988). Radiosensitive human tumour cell lines may not be recovery deficient. *Int. J. Radiat. Biol.*, **54**, 945–953.

PEACOCK, J.H. EADY, J.J., EDWARDS, S., HOLMES, A., MCMILLAN, T.J. & STEEL, G.G. (1989). Initial damage or repair as the major determinant of cellular radiosensitivity? *Int. J. Radiat. Biol.*, **56**, 543–547.

PHILIPSON, L. & LINBERG, U. (1974). Reproduction of adenoviruses. *Comp. Virol.*, **3**, 143–227.

RADFORD, I.R. (1986). Evidence for a general relationship between the induced level of DNA double-strand breakage and cell-killing after X-irradiation of mammalian cells. *Int. J. Radiat. Biol.*, **49**, 611–620.

RAINBOW, A.J. (1974). Repair of radiation-induced DNA breaks in human adenovirus. *Radiat. Res.*, **60**, 155–164.

RAINBOW, A.J. (1980). Reduced capacity to repair irradiated adenovirus in fibroblasts from xeroderma pigmentosum heterozygotes. *Cancer Res.*, **40**, 3945–3949.

RAINBOW, A.J. (1989). Defective repair of UV-damaged DNA in human tumor and SV40-transformed human cells but not in adenovirus-transformed human cells. *Carcinogenesis*, **10**, 1073–1077.

RAINBOW, A.J. (1991). Host cell reactivation of sunlamp-exposed adenovirus in fibroblasts from patients with Bloom's syndrome, ataxia telangiectasia and Huntington's disease. *Env. Mol. Mutagen.*, **17**, 98–103.

RAINBOW, A.J. & HOWES, M. (1979). Decreased repair of gamma-irradiated adenovirus in xeroderma pigmentosum fibroblasts. *Int. J. Radiat. Biol.*, **36**, 621–629.

RAINBOW, A.J. & MAK, S. (1972). DNA strand breakage and biological functions of human adenovirus after gamma irradiation. *Radiat. Res.*, **50**, 319–333.

RAINBOW, A.J. & MAK, S. (1973). DNA damage and biological function of human adenovirus after U.V.-irradiation. *Int. J. Radiat. Biol.*, **24**, 59–72.

RÖSEN, A., TAYLOR, D.M. & DARAI, G. (1987). Influence of ionizing radiation on herpes simplex virus and its genome. *Int. J. Radiat. Biol.*, **52**, 795–804.

SARASIN, A.R. & HANAWALT, P.C. (1978). Carcinogens enhance survival of UV-irradiated simian virus 40 in treated monkey kidney cells: induction of a recovery pathway? *Proc. Natl Acad. Sci. USA*, **75**, 346–350.

SHADLEY, J.D. & WIENCKE, J.K. (1989). Induction of the adaptive response by X-rays is dependent on radiation intensity. *Int. J. Radiat. Biol.*, **56**, 107–118.

SMITH, I.E., COURTENAY, V.D., MILLS, J. & PECKHAM, M.J. (1978). *In vitro* radiation response of cells from four human tumors propagated in immune-suppressed mice. *Cancer Res.*, **38**, 390–392.

TAYLOR, A.M.R. (1978). Unrepaired DNA strand breaks in irradiated ataxia telangiectasia lymphocytes suggested from cytogenetic observations. *Mutat. Res.*, **50**, 407–418.